# Exclusionary School Discipline and School Achievement for Middle and High School Students, by Race and Ethnicity

**DOI:** 10.1001/jamanetworkopen.2023.38989

**Published:** 2023-10-20

**Authors:** Camila Cribb Fabersunne, Seung Yeon Lee, Dannielle McBride, Ali Zahir, Angela Gallegos-Castillo, Kaja Z. LeWinn, Meghan D. Morris

**Affiliations:** 1 Department of Pediatrics, University of California, San Francisco; 2 Department of Pediatrics, Zuckerberg San Francisco General Hospital, San Francisco, California; 3 Independent Researcher; 4 Department of Medicine, University of California, San Francisco; 5 Department of Neurology, University of California, San Francisco; 6 Instituto Familiar de la Raza Inc, San Francisco, California; 7 Department of Psychiatry and Behavioral Health Sciences, University of California, San Francisco; 8 Department of Epidemiology and Biostatistics, University of California, San Francisco

## Abstract

**Importance:**

There are racial and ethnic inequities in exclusionary school discipline (ESD) (ie, a disciplinary action that removes students from their classroom or school environment, eg, referrals, suspensions, and/or expulsions) practices in the US. Exclusionary school discipline has been associated with negative education, health, and criminal justice outcomes.

**Objectives:**

To investigate whether experiencing an ESD event was associated with decreased grade point average (GPA) and whether minoritized students (eg, Black or Latine [description used in database]) are disproportionately affected compared with White students.

**Design, Setting, and Participants:**

In a cohort study using retrospective administrative longitudinal data of children in the 6th to 10th grades (August 18, 2014, to May 26, 2017) in a large, single urban school district in California, linear mixed models were applied to compare the timing of the first exclusionary event and the average change in GPA and evaluate the relative variation among minoritized students experiencing an exclusionary event. Data analysis was conducted from August 18, 2018, to August 21, 2023.

**Exposure:**

Year at which students experienced first ESD events over the study period.

**Main Outcomes and Measures:**

The primary outcome of interest was change in average grade point average (GPA); students’ GPA averaged across courses was averaged across each year.

**Results:**

Of the 16 849 students (8756 [52.0%] male), 21.4% experienced at least 1 ESD event. The mean (SD) age was 14.3 (1.6) years, and 7.5% identified as Black, 25.6% Latine, and 10.0% White. Black and Latine students experienced exclusionary events at nearly 10 and 3 times more than White students (mean [SD]: Black, 6.69 [12.80] events; Latine, 2.01 [6.18] events; White, 0.71 [4.46] events; *P* < .001). When controlling for gender, maternal educational level, race and ethnicity, and school year, having experienced an ESD event in the first year was associated with an average decrease in GPA by 0.88 (95% CI, −0.91 to −0.84) points compared with no ESD events; experiencing ESD events also had significant differences in the second (−0.63 [95% CI, −0.67 to −0.59]) and third (−0.52 [95% CI, −0.57 to −0.47]) years. Black and Latine race and ethnicity was associated with the greatest decrease in GPA compared with White students (Black, −0.56 [95% CI, −0.61 to −0.51]; Latine, −0.51 [95% CI, −0.54 to −0.47]; *P* < .001).

**Conclusion and Relevance:**

This study observed racial and ethnic inequities in ESD prevalence and its association with educational attainment. The findings suggest that it may be beneficial for pediatricians and other health care professionals to screen for exclusion, as experiencing ESD events may affect health across the life course. In addition, it may be useful to categorize ESD events as an adverse childhood experience and abolish the practice from schools as a disciplinary measure.

## Key Points

**Question:**

Is exclusionary school discipline (ESD) associated with grade point average (GPA) in racially and ethnically minoritized students?

**Findings:**

This cohort study evaluated 16 849 middle and high school students from a single large urban district in California over 3 years. Having experienced an ESD event in the first year was associated with a lower average GPA by 0.88 points; Black and Latine students experienced more events and more significantly lower GPA by over half a grade point.

**Meaning:**

The findings of this study suggest there are racial and ethnic disparities in ESD events, and ESD may itself be considered an adverse childhood experience.

## Teaser

This cohort study examines whether experiencing an exclusionary school discipline event is associated with changes in grade point average among middle and high school students.

## Introduction

Exclusionary school discipline (ESD) practices, defined as any discipline that removes students from their classroom or school environment (eg, referrals, suspensions, and/or expulsions), are used across US schools, with 5% to 6% of public school students in the US receiving 1 or more suspensions in 2017-2018.^1,2^ Exclusionary school discipline has increased despite a decrease in school violence and delinquency.^3,4,5^ Studies have shown that ESD does not prevent subsequent behavioral disruptions or create a classroom environment where students feel physically and emotionally safe.^6,7^ ESD may in fact promote both immediate and downstream negative outcomes among affected students. These associated consequences can include poor mental and physical well-being, decreased economic opportunity, increased juvenile justice involvement, and an immediate disruption of a student’s academic trajectory.^8,9^ Exclusionary school discipline can hinder overall school performance through missed instruction, classroom absenteeism, and academic disengagement; the latter can increase attrition and reduce the chance of graduation. Exclusionary school discipline has also been found to increase students’ risk of future classroom disruptions, subsequent ESD events, peer exclusion, and truancy.^10^ Students affected by ESD have a greater risk of disengagement, disrupted social bonds, and misbehavior than those who have not experienced ESD.^4,11,12^ Exclusionary school discipline exposure is also associated with a higher risk of adult incarceration, even when controlling for childhood delinquency.^3,8,13^ Conversely, the importance of educational attainment cannot be overstated. Educational attainment is essential to socioeconomic opportunity, affording individuals access to employment opportunities and higher quality employment, as well as reducing the risk of criminal justice system involvement.^14,15^ Decreased educational attainment also increases one’s risk of poverty and housing instability during both adolescence and adulthood.^16,17^ Consequences further extend to health outcomes—poor educational attainment can lead to psychological impairment and premature mortality.^17,18,19^

Exclusionary school discipline has long featured racial inequities: minoritized students continue to be disproportionately exposed to this form of discipline, further perpetuating racial and ethnic inequities in education and health outcomes.^20^ Black and Latine students are suspended at higher rates compared with White students and receive harsher punishments for similar behaviors.^16,21,22,23^ Experiencing such discriminatory practices falls under the category of an adverse childhood experience (ACE). Many studies have detailed the outcomes of ACEs associated with individuals’ health via chronic stress.^24,25^ Such experiences can result in maladaptive behavior in the short term (eg, impulsiveness, withdrawal, hyperactivity, and aggression) as well as the development of chronic conditions, such as diabetes, asthma, mental illness, and depression (including suicidality).^25^ Such practices, coupled with the fact that minority communities experience a higher incidence of ACEs at baseline due to structural racism affecting minoritized communities, make these practices potentially more harmful.

Despite the enduring effects that ESD can have on a younger person and their life trajectory, most relevant research has only assessed the outcomes of ESD cross-sectionally; few studies have fully delineated the temporality of associated consequences.^26^ Understanding the trajectory-altering outcome of an early ESD event on a student’s educational attainment is needed to understand the magnitude of ESD practices over the life course. In the present study, we accessed multiple years of administrative data from a large urban school district in California to examine the association of ESD with change in students’ grade point average (GPA). We hypothesized that middle- and high-school students receiving ESD would display poorer grades (ie, lower GPA) with racially and ethnically minoritized students disproportionately experiencing more ESD events as well as greater association of those ESD events with grades.

## Methods

Our study sample consisted of administrative data collected between August 18, 2014, and May 26, 2017, on students between grades 6 and 10 in a large urban district in California. This study was deemed exempt by the institutional review board of the University of California, San Francisco, as deidentified administrative data were used in these analyses. The data analysis was performed between August 18, 2018, and August 21, 2023. This study follows Strengthening the Reporting of Observational Studies in Epidemiology (STROBE) reporting guideline for observational studies.

A total of 16 849 students with complete data from 3 consecutive years were included in our analysis. Data included students’ demographic characteristics (age, self-reported gender, self-reported race and ethnicity), family characteristics (maternal educational status), and academic characteristics (special education, school site citizenship, and whether English language and mathematics standards were met). Baseline characteristics were assigned using data from the first year of the study (wave 1).

The primary outcome of interest was average GPA. As provided by the school district, students’ annual GPA values represented the grade point values averaged across courses taken during 2 semesters. The GPA score can range from 0.0 to 4.0.

The primary independent variable of interest was a categorical measure of the year when a student experienced the first ESD event, defined as either a school suspension or disciplinary office referral (ie, a student being removed from the classroom and sent to the administrative office) for each school year. Each student was assigned to 1 of the following 4 categories: (1) no ESD over the study period, (2) the first ESD event during wave 1 (2014-2015), (3) the first ESD event during wave 2 (2015-2016), and (4) the first ESD event during wave 3 (2016-2017). There was substantial overlap in our sample with those who received a suspension and those who received a referral to the office; both captured times that students were removed from the classroom: 84% of students who experienced a suspension also experienced a referral; 25% of students who experienced a referral also experienced a suspension. Our measure of ESD did not include expulsion because the focal school district stopped issuing expulsions before the study period. When discussing ESD throughout this article, we refer to students having experienced an ESD event because this penalty is assigned to a student (rather than being actively sought) and is outside their control.

Because of the consequences of structural racism that create inequities in health and education outcomes, we evaluated differences by self-reported race and ethnicity as a proxy for experiencing racism for those minoritized students. Racial and ethnic categories provided by the district include African American/Black (referred to hereafter as Black), American Indian or Alaskan Native, East Asian (Korean, Japanese, and Chinese), Hispanic/Latine (a/o/x), Other Asian, Pacific Islander (Native Hawaiian, Guamanian, Philippine, Samoan, Laotian, and Other Pacific Islander), South Asian (Asian Indian), Southeast Asian (Cambodian, Hmong, Vietnamese), White (non-Hispanic; referred to hereafter as White), 2 or more races, and unknown race. All students who self-identified as Hispanic/Latine ethnicity and a single racial category were categorized as Latine. Students who self-identified as Latine and 2 or more races were categorized as 2 or more races. We imputed race and ethnicity from second- or third-year data for those who had unknown race or ethnicity in the first year of the study (177 students had imputed racial and ethnic categories). Additional covariates of interest included the following: student’s school grade, gender, maternal educational level, special education enrollment, and school citizenship. School citizenship, together with grades, was designed by the school district to evaluate additional information about a student’s performance in the classroom, such as participation and collaboration with other students. Citizenship scores were tallied across each academic year for each student; a summary variable, ie, proportion excellent, was then created to capture the proportion of a student’s classes in which they received excellence in citizenship. Because of the subjective nature of the citizenship variable, this was not included in the analytic model.

### Statistical Analysis

We used descriptive statistics to examine the distribution of the variables of interest and the longitudinal patterns in average GPA. We performed Kruskal-Wallis analysis of variance tests to evaluate differences among the racial and ethnic groups in the total number of ESD events they experienced over 3 years. To compare the year of the first ESD event with the average GPA, we applied a linear mixed model allowing for repeated measures for GPA across 3 years using participant as a random effect and time as a fixed effect. Including participant as a random effect allowed us to control for any baseline variation in GPA without needing to specifically include the first GPA value as a covariate.

Results are reported as β coefficients responding to the average change in GPA. All variables that attained significance at *P* ≤ .05 with 2-sided testing were considered for inclusion in our multivariable models using a backward stepwise approach (ie, age, gender, race and ethnicity, maternal educational level, and special education). Additionally, a sensitivity analysis was performed using a year of first suspension to understand the association between suspension events and average GPA. All analyses were run using R, version 4.3.1 (R Foundation for Statistical Computing).

## Results

A total of 19 040 students were enrolled in grades 6 to 10 in the focal district between 2014 and 2017; 2062 students (10.8%) were excluded for having not been in the district for each of the 3 consecutive years. An additional 129 students (0.7%) were excluded from analysis due to missing the primary outcome of interest (GPA). The remaining 16 849 students (88.5%) constituted our analytic sample. A comparison of the analytic sample with the excluded students revealed lower representation of tenth graders, males, those with an Individualized Education Plan, and minoritized students (including Black and Latine students) among the analytic sample (eTable 1 in Supplement 1). Those who were excluded from the analysis were older than the analytic sample as fewer had data from grade 6 (12.2% of excluded students had data in the 6th grade compared with 20% of the analytic sample).

Table 1 presents complete descriptive statistics of sample characteristics. The grade level at the beginning of the study was nearly evenly split between the 5 grades (6-10). The mean (SD) age of the students was 14.3 (1.6) years at the beginning of the study period. Slightly more than half (52.0%) were male and 48.0% were female. The racial and ethnic identities of the students were as follows: Black (7.5%), American Indian/Alaskan Native (0.4%), East Asian (36.6%), Latine (25.6%), Pacific Islander (7.1%), South Asian (2.5%), Southeast Asian (3.8%), other Asian (0.4%), White (10.0%), and 2 or more races (2.1%), and 3.9% were unknown. In addition, 12.5% of the students had an Individualized Education Plan indicating a disability.

**Table 1.** Baseline Characteristics of 16 849 Students in Middle and High School Between 2014 and 2017

Variable name	**No. (%)**
Grade level at school year 2014-2015	
6	3373 (20.0)
7	3104 (18.4)
8	3121 (18.5)
9	3709 (22.0)
10	3542 (21.0)
Self-reported gender	
Female	8093 (48.0)
Male	8756 (52.0)
Self-reported race and ethnicity^a^	
African American or Black	1270 (7.5)
American Indian or Alaskan Native	75 (0.4)
East Asian (Korean, Japanese, Chinese)	6165 (36.6)
Hispanic or Latine (a/o)	4311 (25.6)
Other Asian	69 (0.4)
Pacific Islander (Samoan, Guamanian, Philippine (a/o), Native Hawaiian, Laotian, Other Pacific Islander)	1199 (7.1)
South Asian/Asian Indian	416 (2.5)
Southeast Asian (Cambodian, Hmong, Vietnamese)	645 (3.8)
Two or more races	356 (2.1)
White (non-Hispanic)	1680 (10.0)
Unknown	663 (3.9)
Highest maternal educational level noted at baseline	
Graduate school/postgraduate training	712 (4.2)
College graduate	1934 (11.5)
Some college (includes associate degree)	1637 (9.7)
High school graduate	4172 (24.8)
Not a high school graduate	1946 (11.5)
Decline to state	6443 (38.2)
Missing	5 (0.0)
Active IEP, indicating special education	
No	14 749 (87.5)
Yes	2100 (12.5)
Meeting English language arts standards^b^	
Yes	2431 (14.4)
No	6648 (39.5)
Missing	7770 (46.1)
Meeting mathematics standards^b^	
Yes	2860 (17.0)
No	6340 (37.6)
Missing	7649 (45.4)
Proportion of students with excellent citizenship scores^b^	
Proportion of students with excellent scores in ≥75% of their classes	766 (4.5)
Proportion of students with excellent scores in <75% of their classes	15 938 (94.6)
Missing	145 (0.9)
Experiences of ESD	
School year of first experience of ESD	
No ESD events	13 236 (78.6)
2014-2015	1783 (10.6)
2015-2016	1082 (6.4)
2016-2017	748 (4.4)
Any suspension over study period	1041 (6.2)
No. of suspensions, among those with a suspension, median (IQR)	1.0 (1-2)
Total No. of suspensions	
0	15 808 (93.8)
1	646 (3.8)
>1	395 (2.3)
Any referral over study period	3444 (20.4)
No. of referrals, among those with a referral, median (IQR)	2.0 (1-6)
Total No. of referrals	
0	13 405 (79.6)
1	1324 (7.9)
>1	2120 (12.6)
Any ESD event (suspension or referral) over the study period	3613 (21.4)
No. of ESD events among those with an ESD event, median (IQR)	2.0 (1-6)
Total No. of ESD events	
0	13 236 (78.6)
1	1353 (8.0)
>1	2260 (13.4)

Abbreviations: ESD, exclusionary school discipline; IEP, Individualized Education Plan.

^a^ For race and ethnicity, 177 students with unknown race in the first year, race and ethnicity was imputed from subsequent years’ individual data.

^b^ Citizenship, meeting English language arts standards, and meeting mathematics standards are included for descriptive information; these were not covariates of interest and thus were not included in the multivariable models.

Over the study period, 3613 students (21.4%) experienced an ESD event. Among them, most (3444 [95.3%] students) received at least 1 office referral over the study period (range, 1-132; IQR, 1-6), and 1041 (28.8%) received at least 1 suspension over the study period (range, 1-18; IQR, 1-2). Of students who experienced a suspension, 872 (83.8%) also experienced at least 1 office referral. Most students’ first experiences of ESD took place in the first year: 1783 students (10.6%) experienced the first ESD event in year 1 (2014), 1082 (6.4%) first experienced the first ESD event in year 2, and 748 (4.4%) experienced their first ESD event in year 3.

Black students experienced the highest average number of ESD events of any racial or ethnic category by nearly10 times compared with White students (mean [SD]: Black, 6.69 [12.80] events; White, 0.71 [4.46] events), with students of 2 or more races experiencing the next highest with a mean (SD) of 2.32 (8.71) events. Latine students experienced 2.01 (16.18) events, American Indian/Alaskan Native students experienced 1.93 (5.33) events, and Pacific Islander students experienced 1.16 (5.39) events. Southeast Asian and East Asian students experienced the fewest events (mean [SD]: Southeast Asian, 0.30 [1.57] events; East Asian, 0.18 [1.43] events; *P* < .001). The Figure shows the patterns in annual GPA and the timing of the first ESD event for all students and for students whose first ESD event was in year 1.

**Figure.**
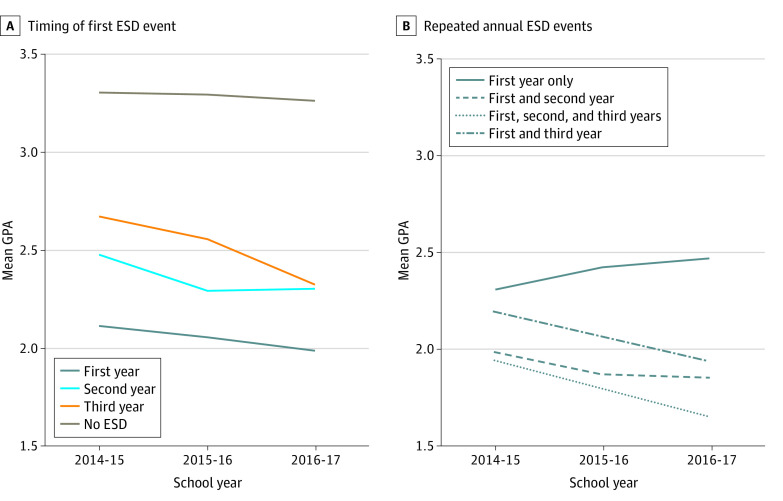
Patterns in Annual Average Grade Point Average (GPA) A, Timing of first exclusionary school discipline (ESD) event: among all students, pattern of average GPA by year of first ESD event. B, Repeat annual ESD events: among students whose first ESD was in year 1 (n = 1783).

To evaluate the individual association between an ESD event and change in student’s average GPA, we ran one generalized linear mixed model adjusting for gender, maternal educational level, race and ethnicity, and school year (Table 2). Having experienced an ESD event in the first year (2014) of the study was associated with an average GPA decrease of 0.88 grade points (95% CI, −0.91 to −0.84). Experiencing an ESD event in the second and third years was similarly significant, with smaller associated decreases: −0.63 (95% CI, −0.67 to −0.59) for 2015 and −0.52 (95% CI, −0.57 to −0.47) for 2016. Minoritized students experienced an associated decrease in average GPA even when controlling for these ESD events, with Black and Latine students having an association of over a half-point decrease in average GPA (Black, −0.56; 95% CI, −0.61 to −0.51; Latine, −0.51; 95% CI, −0.54 to −0.47).

**Table 2.** Bivariate and Multivariable Mixed Linear Models of Select Baseline Characteristics and GPA Across 3 Years

Variable name	Coefficient (bivariate)	*P* value	Coefficient (multivariable)	*P* value
School year of first ESD				
No ESD	1 [Reference]	NA	1 [Reference]	NA
2014-2015	−1.24 (−1.26 to −1.21)	<.001	−0.88 (−0.91 to −0.84)	<.001
2015-2016	−0.93 (−0.96 to −0.90)	<.001	−0.63 (−0.67 to −0.59)	<.001
2016-2017	−0.77 (−0.81 to −0.74)	<.001	−0.52 (−0.57 to −0.47)	<.001
Total suspensions, over study period				
0	1 [Reference]	NA	NA	NA
1	−1.12 (−1.16 to −1.08)	<.001	NA	NA
>1	−1.51 (−1.57 to −1.46)	<.001	NA	NA
Total referrals, over the study period				
0	1 [Reference]	NA	NA	NA
1	−0.68 (−0.71 to −0.65)	<.001	NA	NA
>1	−1.25 (−1.27 to −1.23)	<.001	NA	NA
Total ESD events, over the study period				
0	1 [Reference]	NA	NA	NA
1	−0.69 (−0.71 to −0.66)	<.001	NA	NA
>1	−1.26 (−1.28 to −1.24)	<.001	NA	NA
Age	−0.08 (−0.09 to −0.08)	<.001	−0.09 (−0.10 to −0.09)	<.001
Race/ethnicity				
African American or Black	−1.06 (−1.10 to −1.03)	<.001	−0.56 (−0.61 to −0.51)	<.001
American Indian or Alaskan Native	−0.68 (−0.80 to −0.57)	<.001	−0.45 (−0.60 to −0.30)	<.001
Asian Indian	−0.13 (−0.18 to −0.08)	<.001	−0.02 (−0.09 to 0.05)	.28
East Asian	0.11 (0.08 to 0.13)	<.001	0.16 (0.12 to 0.20)	<.001
Hispanic or Latine	−0.81 (−0.84 to −0.78)	<.001	−0.51 (−0.54 to −0.47)	<.001
Other Asian	−0.11 (−0.23 to 0.00)	.05	0.01 (−0.14 to 0.17)	.44
Pacific Islander	−0.33 (−0.37 to −0.29)	<.001	−0.20 (−0.25 to −0.15)	<.001
Southeast Asian	0.04 (−0.00 to 0.09)	.06	0.11 (0.05 to 0.17)	<.001
White	1 [Reference]		1 [Reference]	
Two or more races	−0.26 (−0.32 to −0.20)	<.001	−0.13 (−0.21 to −0.05)	.001
Unknown	−0.05 (−0.09 to −0.01)	.01	−0.00 (−0.06 to 0.05)	.43
Gender (self-identified)				
Female	1 [Reference]	NA	1 [Reference]	NA
Male	−0.33 (−0.34 to −0.31)	<.001	−0.20 (−0.22 to −0.18)	<.001
Highest maternal educational level noted at baseline				
Graduate school/postgraduate training	1 [Reference]	NA	1 [Reference]	NA
College graduate	−0.16 (−0.21 to −0.12)	<.001	−0.09 (−0.15 to −0.04)	.001
Some college (includes associate degree)	−0.54 (−0.59 to −0.50)	<.001	−0.24 (−0.30 to −0.18)	<.001
High school graduate	−0.55 (−0.59 to −0.50)	<.001	−0.29 (−0.34 to −0.23)	<.001
Not a high school graduate	−0.64 (−0.69 to −0.60)	<.001	−0.35 (−0.41 to −0.29)	<.001
Decline to state	−0.67 (−0.72 to −0.63)	<.001	−0.29 (−0.35 to −0.24)	<.001
Active IEP indicating special education				
No	1 [Reference]	NA	1 [Reference]	NA
Yes	−0.60 (−0.62 to −0.58)	<.001	−0.17 (−0.20 to −0.14)	<.001
School year				
2014-15	1 [Reference]	NA	1 [Reference]	NA
2015-16	−0.03 (−0.05 to −0.01)	.008	0.05 (0.04 to 0.07)	<.001
2016-17	−0.07 (−0.08 to −0.05)	<.001	0.09 (0.08 to 0.11)	<.001

Abbreviations: ESD, exclusionary school discipline; GPA, grade point average; IEP, Individualized Education Plan; NA, not applicable.

eTable 2 in Supplement 1 reports the results of the sensitivity analysis. We constructed a similar categorical exposure variable indicating, for each student, the year during which the first suspension occurred. There are similar associations of decreased average GPA for exposures across all 3 years, with a similar pattern as the main result (a decrease of −0.88 points; 95% CI, −0.94 to −0.82 in 2014; −0.84 points; 95% CI, −0.92 to −0.76 in 2015; and −0.71; 95% CI, −0.76 to −0.63 in 2016).

## Discussion

Our findings suggest that experiencing an ESD event was associated with a decrease in a student’s average GPA resulting in a lower average GPA over time and that minoritized students, specifically Black and Latine, had an additional associated decrease in comparison with their White counterparts. Overall, we observed an associated decrease of more than half a grade point (0.88 in the first year, 0.63 in the second year, and 0.52 in the third year) on average among students who received an ESD event. Students who experienced an ESD event earlier in the study period had more significant decreases in their average GPA compared with those who experienced their first ESD event later in the study period. This pattern may have emerged because classroom removal initiates a cascade of events that impair a student’s ability to succeed academically, ultimately leading to decreased academic performance. Our findings are worrisome because GPA is a common gateway metric influencing students’ opportunities to participate in extracurricular activities, maintain eligibility for academic scholarships, and be considered for high school graduation and college admission.^3,27^

We also noted clear disparities in ESD event rates and the association of decreased average GPA for minoritized students, particularly Black and Latine students. Our results are consistent with the literature, which has reported racial and ethnic inequities in ESD events nationwide, with a similar pattern of Black and Latine students having the most significant rates of ESD, as well as a gap in educational attainment.^28^ Several theories may explain this association. Differential selection and processing hypothesize that minoritized students are more likely to be targeted for wrongdoing and thus more apt to be differentially selected for disciplinary consequences.^16,29^ Furthermore, ESD events are not singular occurrences but rather multifaceted processes that involve student behavior as well as the beliefs of school staff and institutional policies.

Exclusionary school discipline is therefore fraught with bias (ie, structural racism) that disadvantages minoritized students.^16,22^ Student behavior can often be a manifestation of trauma faced outside the classroom. The resultant symptoms of trauma (eg, hyperactivity, impulsiveness) are commonly labeled misbehavior—either due to biases or uninformed perceptions about manifestations of trauma—and met with exclusion from schools. The identification of so-called misbehavior is more common among students who are targeted by racism (ie, not White). On average, minoritized youth (eg, Black and Latine) have higher rates of ACEs because of structural racism’s traumatic impact on a youth’s community and family.^30^ For example, in the Black community, there are higher rates of witnessing neighborhood or interpersonal (eg, racialized police brutality, mass incarceration secondary to racist laws targeting minoritized communities) violence, having an incarcerated family member, living in an under-resourced neighborhood, and experiencing racism or poverty.^30,31^

Taken together, the legacy of racism in the Black and Latine communities has lasting effects in educational attainment and exclusionary school disciplinary practices. We acknowledge that race is a social construct and that systemic racism affects different people from various self-identified racial and/or ethnic groups differently.^32^ Exploring the effect of ESD on specific racial and ethnic groups can inform interventions to interrupt the legacy of racism that has affected students differentially.

Our study is consistent with other literature that reports an association with a lower educational attainment after experiencing an ESD event.^10^ Exclusionary school discipline is similar to ACEs—both are traumatic events during a youth’s development that can have a lasting effect on one’s life.^25,33^ Our analysis supports the consideration that ESD is a trajectory-altering effect leading to decreased educational attainment (GPA in this study) and very well may deleteriously affect mental and physical health outcomes. Considering the myriad of health consequences to which academic failure can lead, it is important that pediatricians implement appropriate assessments, interventions, and prevention measures to address school exclusion.^34^ In light of the multifaceted and enduring downstream effects of ESD events, we recommend that ESD be categorized as an ACE to allow for assessment and intervention of this trauma in clinical settings and further examine its impact across the life course.

The American Academy of Pediatrics has previously encouraged pediatricians to take a more active role in assisting students and families who have experienced ESD and has recommended that clinicians encourage schools to adopt alternative (nonexclusionary) policies to address student behavior.^35^ Pilot programs have observed some success with alternative disciplinary practices. For example, the Quiet Time program,^36^ which introduces meditation or other quiet, centering activities into students’ days, has been reported to reduce school violence and ESD and narrow the African American achievement gap.^37^ As another alternative, schools could pivot from punitive discipline to restorative justice practices with emphases on problem solving and cooperation to repair and prevent harm (eg, peer or restorative circles, student-teacher discussions).^38^ Additionally, we advocate for school-wide positive behavior interventions that institute trauma-informed care and are intended to strengthen social bonds within the school and broader community. These positive relationship outcomes can be realized by focusing on trauma-informed principles: establishing systems that promote safety, offering predictable routines and social interactions, fostering a calm physical environment, providing transparent rules and nonpunitive consequences for rule violations, teaching social-emotional skills, eliciting students’ input on school policies, and encouraging family and community involvement, including support for families on parenting or stress management.^39,40^ Finally, we recommend that policy makers at the state and federal levels prioritize replacing ESD practices (including elimination of school police presence) with in-school behavioral and mental health supports.

### Limitations

We acknowledge that our study has several limitations. First, in our analyses, we were unable to control for many individual, school, and neighborhood effects. The school a child attends and the neighborhood where a student lives influence academic success, yet these variables were not accounted for in our analyses. These omissions may have led to bias toward the null and a muted observed association between ESD and student GPA. Second, our data allow a 3-year snapshot of the association between experiencing an ESD and a decrease in annual average GPA but do not allow for examination of longer-term impacts of ESD on GPA or subsequent educational attainment measures (high school diploma or higher education certification). Similarly, we were unable to account for a history of ESD before the study period. Third, compared with the analytic sample, there were differences in those who had incomplete data and thus were excluded from the analysis (eTable 1 in Supplement 1). Our results may underestimate the association of ESD with average GPA for those who are minoritized, such as Black and Latine students. Fourth, we were unable to account for chronic (nonexclusionary) absenteeism, which has been correlated with decreasing educational attainment.^41^ Fifth, due to the retrospective cohort study design, causality is not assessed in these models. Sixth, our study population was from a single, large urban school district in California, which limits the generalizability to other school districts with different policies and school climates.

## Conclusions

Our study involved a retrospective analysis of a middle- and high-school cohort in a single large, urban school district in California that observed racial disparities in the prevalence of ESD and its association with educational attainment (GPA). This research study just skims the surface of understanding the traumatic effect that ESD can have on a young person’s educational outcomes, and further research is needed to better understand the multifaceted nature of the impact of an ESD event. We suggest that more longitudinal and prospective studies be performed to further evaluate the numerous downstream effects of ESD. Subsequent studies should incorporate qualitative methods to examine the experiences of younger students who have experienced ESD; the student perspective has yet to be consistently considered, and interviews with students can reveal additional aspects of ESD not captured in the existent literature. Lastly, we encourage scholars to pursue a clearer understanding of the association of ESD with clinical outcomes and how screening for ESD as an ACE can be implemented in clinical practice.

## Notes

Supplement 1. **eTable 1.** Baseline Characteristics of Students in Middle and High School Between 2014-2017, Analytic Sample Compared to Excluded Sample **eTable 2.** Bivariate and Multivariable Mixed Linear Models of Select Baseline Characteristics and GPA Across Three Years, Using Year of First Suspension

Supplement 2.
Data Sharing Statement

## References

[1] US Dept of Education. Suspensions and expulsions in public schools: 2017-18 Civil Rights data collection. May 2021. Accessed September 15, 2023. https://ocrdata.ed.gov/assets/downloads/Suspensions_and_Expulsion_Part2.pdf

[2] Losen DJ, Whitaker A. 11 Million days lost: race, discipline, and safety at US public schools. 2018. Accessed September 15, 2023. https://www.aclu.org/wp-content/uploads/legal-documents/final_11-million-days_ucla_aclu.pdf

[3] Fabelo T, Thompson MD, Plotkin M, Carmichael D, Marchbanks MPI, Booth EA. Breaking schools’ rules: a statewide study of how school discipline relates to students’ success and juvenile justice involvement. July 2011. Accessed September 15, 2023. https://csgjusticecenter.org/wp-content/uploads/2020/01/Breaking_Schools_Rules_Report_Final.pdf

[4] Skiba RJ, Arredondo MI, Williams NT. More than a metaphor: the contribution of exclusionary discipline to a school-to-prison pipeline. Equity Excell Educ. 2014;47(4):546-564. doi:10.1080/10665684.2014.958965

[5] Duarte CD, Moses C, Brown M, . Punitive school discipline as a mechanism of structural marginalization with implications for health inequity: a systematic review of quantitative studies in the health and social sciences literature. Ann N Y Acad Sci. 2023;1519(1):129-152. doi:10.1111/nyas.14922 36385456 PMC10929984

[6] Massar MM, McIntosh K, Eliason BM. Do out-of-school suspensions prevent future exclusionary discipline? Positive Behavioral Interventions & Supports. May 2015. Accessed September 15, 2023. https://global-uploads.webflow.com/5d3725188825e071f1670246/5d79778ee21ac97f0bfeb9a6_evalbrief_may2015.pdf

[7] Steinberg M, Allensworth E, Johnson DW. Student and teacher safety in Chicago public schools: the roles of community context and school social organization. May 2011. Accessed September 15, 2023. https://files.eric.ed.gov/fulltext/ED519414.pdf

[8] Wolf KC, Kupchik A. School suspensions and adverse experiences in adulthood. Justice Q. 2016;8825(April):1-24.

[9] Gakidou E, Cowling K, Lozano R, Murray CJ. Increased educational attainment and its effect on child mortality in 175 countries between 1970 and 2009: a systematic analysis. Lancet. 2010;376(9745):959-974. doi:10.1016/S0140-6736(10)61257-3 20851260

[10] Chu EM, Ready DD. Exclusion and urban public high schools: short- and long-term consequences of school suspensions. Am J Educ. 2018;124(4). doi:10.1086/698454

[11] Mankovich A, Christopher A. Mallett: the school-to-prison pipeline: a comprehensive assessment. J Youth Adolesc. Published online 2016

[12] Nicholson-Crotty S, Birchmeier Z, Valentine D. Exploring the impact of school discipline on racial disproportion in the juvenile justice system. Soc Sci Q. 2009;90(4):777-1038. doi:10.1111/j.1540-6237.2009.00674.x

[13] Rosenbaum JE. Educational and criminal justice outcomes 12 years after school suspension. Youth Soc. 2020;52(4):515-547. doi:10.1177/0044118X17752208 32528191 PMC7288849

[14] Lleras-Muney A. The relationship between education and adult mortality in the United States. Rev Econ Stud. 2005;72(1):189-221. doi:10.3386/w8986

[15] Lochner L, Moretti E. The effect of education on crime: Evidence from prison inmates, arrests, and self-reports. Am Econ Rev. 2004;94(1):155-189. doi:10.1257/000282804322970751

[16] Gregory A, Skiba RJ, Noguera PA. The achievement gap and the discipline gap: two sides of the same coin? Educ Res. 2010;39(1):59-68. doi:10.3102/0013189X09357621

[17] Sum A, Khatiwada I, McLaughlin J, Palma S. The consequences of dropping out of high school. Center for Labor Market Studies. October 1, 2009. Accessed September 22, 2023. https://search.issuelab.org/resource/the-consequences-of-dropping-out-of-high-school-joblessness-and-jailing-for-high-school-dropouts-and-the-high-cost-for-taxpayers.html#:~:text=state%2C%20or%20country-,The%20Consequences%20of%20Dropping%20Out%20of%20High%20School%3A%20Joblessness%20and,the%20High%20Cost%20for%20Taxpayers&text=Dropping%20out%20of%20high%20school%20is%20correlated%20with%20lower%20employment,this%20research%20paper%27s%20data%20analysis.

[18] Hummer RA, Hernandez EM. The effect of educational attainment on adult mortality in the United States. Popul Bull. 2013;68(1):1-16. 25995521 PMC4435622

[19] Maynard BR, Salas-Wright CP, Vaughn MG. High school dropouts in emerging adulthood: substance use, mental health problems, and crime. Community Ment Health J. 2015;51(3):289-299. doi:10.1007/s10597-014-9760-5 25030805 PMC4655594

[20] González T, Etow A, De La Vega C. Health equity, school discipline reform, and restorative justice. J Law Med Ethics. 2019;47(2 suppl):47-50. doi:10.1177/1073110519857316 31298124

[21] Schollenberger TL. Racial disparities in school suspension and subsequent outcomes. In: Losen DJ, ed. Closing the School Discipline Gap. Teachers College Press; 2015.

[22] Morris EW, Perry BL. The punishment gap: school suspension and racial disparities in achievement. Soc Probl. 2016;63(1):68-86. doi:10.1093/socpro/spv026

[23] Walker BLT. “Loud, proud, and love a crowd:” African American girls and school discipline practices. Middle Sch J. 2020;51(1):12-18. doi:10.1080/00940771.2019.1689776

[24] Kerker BD, Zhang J, Nadeem E, . Adverse childhood experiences and mental health, chronic medical conditions, and development in young children. Acad Pediatr. 2015;15(5):510-517. doi:10.1016/j.acap.2015.05.005 26183001 PMC4562867

[25] Felitti VJ, Anda RF, Nordenberg D, . Relationship of childhood abuse and household dysfunction to many of the leading causes of death in adults: the Adverse Childhood Experiences (ACE) study. Am J Prev Med. 1998;14(4):245-258. doi:10.1016/S0749-3797(98)00017-8 9635069

[26] Andrew M, Blake MK. The long arm of early exclusionary school discipline? a multi-model analysis. Youth Soc. 2021;55(2):238-258. doi:10.1177/0044118X211042643

[27] Pufall Jones E, Margolius M, Rollock M, Tang Yan C, Cole ML, Zaff JF. Disciplined and disconnected: how students experience exclusionary discipline in Minnesota and the promise of non-exclusionary alternatives. June 2018. Accessed September 15, 2023. https://files.eric.ed.gov/fulltext/ED586336.pdf

[28] Stanford Center for Education Policy Analysis. Racial and ethnic achievement gaps. Accessed September 15, 2023. https://cepa.stanford.edu/educational-opportunity-monitoring-project/achievement-gaps/race/

[29] Piquero AR. Disproportionate minority contact. Future Child. 2008;18(2):59-79. doi:10.1353/foc.0.0013 21337998

[30] Slopen N, Shonkoff JP, Albert MA, . Racial disparities in child adversity in the US: interactions with family immigration history and income. Am J Prev Med. 2016;50(1):47-56. doi:10.1016/j.amepre.2015.06.013 26342634

[31] Hunt TKA, Slack KS, Berger LM. Adverse childhood experiences and behavioral problems in middle childhood. Child Abuse Negl. 2017;67:391-402. doi:10.1016/j.chiabu.2016.11.005 27884508 PMC5436949

[32] Flanagin A, Frey T, Christiansen SL; AMA Manual of Style Committee. Updated guidance on the reporting of race and ethnicity in medical and science journals. JAMA. 2021;326(7):621-627. doi:10.1001/jama.2021.13304 34402850

[33] Merrick MT, Ford DC, Ports KA, . Vital signs: estimated proportion of adult health problems attributable to adverse childhood experiences and implications for prevention—25 states, 2015-2017. MMWR Morb Mortal Wkly Rep. 2019;68(44):999-1005. doi:10.15585/mmwr.mm6844e1 31697656 PMC6837472

[34] Byrd RS. School failure: assessment, intervention, and prevention in primary pediatric care. Pediatr Rev. 2005;26(7):233-243. Published online 2005. doi:10.1542/pir.26.7.233 15994993

[35] Lamont JH, Devore CD, Allison M, et al; Council on School Health. Out-of-school suspension and expulsion. Pediatrics. 2013;131(3):e1000–e1007.

[36] Contractor D, Staats C. Interventions to address racialized discipline disparities and school “push out.” Kirwan Institute Policy Brief; May 1, 2014. Accessed September 22, 2023. https://search.issuelab.org/resource/interventions-to-address-racialized-discipline-disparities-and-school-push-out.html

[37] Center for Wellness and Achievement in Education. Quiet Time Program Report. April 21, 2015. Accessed September 15, 2023. https://cdn.theatlantic.com/assets/media/files/quiet-time-white-paper.pdf

[38] Sumner MD, Silverman CJ, Frampton ML. School-based restorative justice as an alternative to zero-tolerance policies: lessons from West Oakland. Accessed September 15, 2023. https://www.law.berkeley.edu/files/thcsj/10-2010_School-based_Restorative_Justice_As_an_Alternative_to_Zero-Tolerance_Policies.pdf

[39] Reinbergs EJ, Fefer SA. Addressing trauma in schools: Multitiered service delivery options for practitioners. Psychol Sch. 2018;55(3):250-263. doi:10.1002/pits.22105

[40] Welsh RO, Little S. The school discipline dilemma: a comprehensive review of disparities and alternative approaches. Rev Educ Res. 2018;88(5):752-794. doi:10.3102/0034654318791582

[41] Keppens G. School absenteeism and academic achievement: does the timing of the absence matter? Learn Instr. Accessed September 20, 2023 doi:10.1016/j.learninstruc.2023.101769

