# Supplementary material for: Exclusionary School Discipline and School Achievement for Middle and High School Students, by Race and Ethnicity
Source: JAMA Netw Open. 2023 Oct 20;6(10):e2338989. doi: 10.1001/jamanetworkopen.2023.38989 (PMC10589805; doi:10.1001/jamanetworkopen.2023.38989)
Supplement: Supplement 1. — eTable 1. Baseline Characteristics of Students in Middle and High School Between 2014-2017, Analytic Sample Compared to Excluded Sample eTable 2. Bivariate and Multivariable Mixed Linear Models of Select Baseline Characteristics and GPA Across Three Years, Using Year of First Suspension [file jamanetwopen-e2338989-s001.pdf]

Supplementary Online Content

Cribb Fabersunne C, Lee SY, McBride D, et al. Exclusionary school discipline and school achievement for middle and high school students, by race and ethnicity. *JAMA Netw Open*. 2023;6(10):e2338989. doi:10.1001/jamanetworkopen.2023.38989

eTable 1. Baseline Characteristics of Students in Middle and High School Between 2014-2017, Analytic Sample Compared to Excluded Sample

eTable 2. Bivariate and Multivariable Mixed Linear Models of Select Baseline Characteristics and GPA Across Three Years, Using Year of First Suspension

This supplementary material has been provided by the authors to give readers additional information about their work.

© 2023 Cribb Fabersunne C et al. *JAMA Network Open.*

eTable 1. Baseline characteristics of students in middle and high school between 2014-2017, analytic sample compared to excluded sample

Variable	Frequency		p-value
	N (%) or median (IQR)		
	Analytic Sample (N= 16849)	Excluded Sample (N=2191)	
**Grade level at school year 2014-2015**			<0.001
6	3373 (20.0)	267 (12.2)	
7	3104 (18.4)	468 (21.4)	
8	3121 (18.5)	502 (22.9)	
9	3709 (22.0)	431 (19.7)	
10	3542 (21.0)	523 (23.9)	
**Self-reported gender**			0.215
Female	8093 (48.0)	1021 (46.6)	
Male	8756 (52.0)	1170 (53.4)	
**Self-reported race/ethnicity**			p<0.001
African American or Black	1270 (7.5)	350 (16.0)	
American Indian or Alaskan Native	75 (0.4)	19 (0.9)	
East Asian (Korean, Japanese, Chinese)	6165 (36.6)	200 (9.1)	
Hispanic or Latine(a/o)	4311 (25.6)	745 (34.0)	
Other Asian	69 (0.4)	5 (0.2)	
Pacific Islander (Samoan, Guamanian, Phillipine (a/o), Native Hawaiian, Laotian and other Pacific Islander)	1199 (7.1)	134 (6.1)	
South Asian / Asian Indian	416 (2.5)	45 (2.1)	
Southeast Asian (Cambodian, Hmong, Vietnamese)	645 (3.8)	42 (1.9)	
Two or More Races	356 (2.1)	2 (0.1)	
White (Non Hispanic)	1680 (10.0)	329 (15.0)	
Unknown	663 (3.9)	320 (14.6)	
**Highest maternal education noted at baseline**			<0.001
Graduate school/postgraduate training	712 (4.2)	148 (6.8)	
College graduate	1934 (11.5)	251 (11.5)	
Some college (includes AA degree)	1637 (9.7)	226 (10.3)	
High school graduate	4172 (24.8)	307 (14.0)	
Not a high school graduate	1946 (11.5)	150 (6.8)	
Decline to state	6443 (38.2)	1107 (50.5)	
missing	5 (0.0)	0 (0.0)	

© 2023 Cribb Fabersunne C et al. *JAMA Network Open.*

**Active IEP, indicating special education**			0.027
No	14749 (87.5)	1882 (85.9)	
Yes	2100 (12.5)	309 (14.1)	
**Meeting English Language Arts (ELA) standards***			<0.001
Yes meeting standards	2431 (14.4)	215 (9.8)	
Not meeting standards	6648 (39.5)	674 (30.8)	
Missing	7770 (46.1)	1302 (59.4)	
**Meeting Math standards***			<0.001
Yes meeting standards	2860 (17.0)	319 (14.6)	
Not meeting standards	6340 (37.6)	605 (27.6)	
Missing	7649 (45.4)	1267 (57.8)	
**Proportion of students "Citizenship Scores" that are excellent***			<0.001
Proportion of students with excellent scores in >= 75% of their classes	766 (4.5)	63 (2.9)	
Proportion of students with excellent scores in < 75% of their classes	15938 (94.6)	1777 (81.1)	
Missing	145 (0.9)	351 (16.0)	
**Experiences of exclusionary school discipline (ESD)**			
**School year of first experience of ESD**			0.213
no ESD events	13236 (78.6)	1751 (79.9)	
2014-2015	1783 (10.6)	360 (16.4)	
2015-2016	1082 (6.4)	79 (3.6)	
2016-2017	748 (4.4)	1 (0.1)	
**Any suspension over study period**	1041 (6.2)	127 (5.8)	0.513
**Median number of suspensions, among those with a suspension**	1.0 (IQR=1.0)	1.0 (IQR=1.0)	
**Total number of suspensions**			
0	15808 (93.8)	2085 (95.2)	<0.001
1	646 (3.8)	77 (3.5)	
>1	395 (2.3)	29 (1.3)	
**Any referral over study period**	3444 (20.4)	389 (17.8)	0.003
**Median number of referrals, among those with a referral**	2.0 (IQR = 5)	2.0 (IQR = 4)	
**Total number of referrals**			
0	13405 (79.6)	1882 (85.9)	<0.001
1	1324 (7.9)	157 (7.2)	
>1	2120 (12.6)	152 (6.9)	

© 2023 Cribb Fabersunne C et al. *JAMA Network Open.*

**Any ESD (suspension or referral), over the study period**	3613 (21.4)	440 (20.1)	0.151
**Median number of ESD events, among those with an ESD event**	2.0 (IQR = 5)	2.0 (IQR = 4)	
**Total number of ESD**			<0.001
0	13236 (78.6)	1751 (79.9)	
1	1353 (8.0)	205 (9.4)	
>1	2260 (13.4)	235 (10.7)	

*Citizenship, Meeting ELA and Math standards are included in Table 1 for descriptive information; these are not co-variates of interest and thus are not included in our multivariable models

© 2023 Cribb Fabersunne C et al. *JAMA Network Open.*

eTable 2. Bivariate and multivariable mixed linear models of select baseline characteristics and GPA across three years, using year of first suspension

Variable name		Coefficient (bivariate)	Coefficient (multivariable)
School year of first suspension	No Suspension	-	
	2014-2015	-1.36 (-1.40 to -1.31, p<0.001)	-0.88 (-0.94--0.82, p<0.001)
	2015-2016	-1.27 (-1.33 to -1.21, p<0.001)	-0.84 (-0.92--0.76, p<0.001)
	2016-2017	-1.12 (-1.18 to -1.06, p<0.001)	-0.71 (-0.79--0.63, p<0.001)
Age		-0.08 (-0.09 to -0.08, p<0.001)	-0.08 (-0.09--0.08, p<0.001)
Race/ethnicity	White (*reference*)	-	-
	African American	-1.06 (-1.10 to -1.03, p<0.001)	-0.71 (-0.76--0.66, p<0.001)
	American Indian or Alaskan Native	-0.68 (-0.80 to -0.57, p<0.001)	-0.54 (-0.70--0.39, p<0.001)
	Asian Indian	-0.13 (-0.18 to -0.08, p<0.001)	-0.03 (-0.10-0.04, p=0.216)
	East Asian	0.11 (0.08 to 0.13, p<0.001)	0.21 (0.17-0.25, p<0.001)
	Hispanic or Latino	-0.81 (-0.84 to -0.78, p<0.001)	-0.59 (-0.63--0.55, p<0.001)
	Other Asian	-0.11 (-0.23 to 0.00, p=0.053)	0.02 (-0.15-0.18, p=0.419)
	Pacific Islander	-0.33 (-0.37 to -0.29, p<0.001)	-0.19 (-0.25--0.14, p<0.001)

© 2023 Cribb Fabersunne C et al. *JAMA Network Open.*

	Southeast Asian	0.04 (-0.00 to 0.09, p=0.057)	0.15 (0.08-0.21, p<0.001)
	Two or More Races	-0.26 (-0.32 to -0.20, p<0.001)	-0.15 (-0.23--0.07, p<0.001)
	Unknown	-0.05 (-0.09 to -0.01, p=0.013)	0.00 (-0.05-0.06, p=0.478)
Gender (self-identified)	Female	-	-
	Male	-0.33 (-0.34 to -0.31, p<0.001)	-0.25 (-0.27--0.23, p<0.001)
Maternal highest education	Graduate school/postgraduate training	-	-
	College graduate	-0.16 (-0.21 to -0.12, p<0.001)	-0.12 (-0.18--0.06, p<0.001)
	Some college (includes associate degree)	-0.54 (-0.59 to -0.50, p<0.001)	-0.29 (-0.35--0.23, p<0.001)
	High school graduate	-0.55 (-0.59 to -0.50, p<0.001)	-0.33 (-0.38--0.27, p<0.001)
	Not a high school graduate	-0.64 (-0.69 to -0.60, p<0.001)	-0.41 (-0.47--0.35, p<0.001)
	Decline to state	-0.67 (-0.72 to -0.63, p<0.001)	-0.34 (-0.40--0.29, p<0.001)
Active IEP indicating special education	No	-	-
	Yes	-0.60 (-0.62 to -0.58, p<0.001)	-0.19 (-0.21--0.16, p<0.001)
School Year	2014-15	-	-
	2015-16	-0.03 (-0.05 to -0.01, p=0.008)	0.05 (0.03-0.06, p<0.001)

© 2023 Cribb Fabersunne C et al. *JAMA Network Open.*

	2016-17	-0.07 (-0.08 to -0.05, p<0.001)	0.08 (0.06-0.10, p<0.001)

© 2023 Cribb Fabersunne C et al. *JAMA Network Open.*
