# Supplementary material for: Exclusionary School Discipline and School Achievement for Middle and High School Students, by Race and Ethnicity
Source: JAMA Netw Open. 2023 Oct 20;6(10):e2338989. doi: 10.1001/jamanetworkopen.2023.38989 (PMC10589805; doi:10.1001/jamanetworkopen.2023.38989)
Supplement: Supplement 2. — Data Sharing Statement [file jamanetwopen-e2338989-s002.pdf]

Data Sharing Statement

Cribb Fabersunne. Exclusionary School Discipline and School Achievement for Middle and High School Students, by Race and Ethnicity. *JAMA Netw Open*. Published October 20, 2023. doi:10.1001/jamanetworkopen.2023.38989

Data
Data available: No
